# Differential pro-apoptotic effects of synthetic 4-thiazolidinone derivative Les-3288, doxorubicin and temozolomide in human glioma U251 cells

**DOI:** 10.3325/cmj.2017.58.150

**Published:** 2017-04

**Authors:** Lesya I. Коbylinska, Olga Yu. Klyuchivska, Iryna I. Grytsyna, Natalia Finiuk, Rostyslav R. Panchuk, Marina O. Starykovych, Lilya Lehka, Roman B. Lesyk, Borys S. Zіmenkovsky, Rostyslav S. Stoika

**Affiliations:** 1 Department of Biochemistry, Danylo Halytsky Lviv National Medical University, Lviv, Ukraine; 2 Department of Pharmaceutical, Organic and Bioorganic Chemistry, Danylo Halytsky Lviv National Medical University, Lviv, Ukraine; 3 Department of Regulation of Cell Proliferation and Apoptosis, Institute of Cell Biology, Lviv, Ukraine

## Abstract

**Aim:**

To compare various pro-apoptotic effects of synthetic 4-thiazolidinone derivative (Les-3288), doxorubicin (Dox) and temozolomide (TMZ) in the treatment of human glioma U251 cells to improve treatment outcomes of glioblastoma and avoid anticancer drug resistance.

**Methods:**

The cytotoxic effects of drugs used in human glioma U251 cells were measured by cell viability and proliferation assay (MTT), Trypan blue exclusion test, and Western-blot analysis of the apoptosis-related proteins. In addition, flow cytometry study of reactive oxygen species (ROS) level in glioma cells was carried out. Cytomorphological changes in treated cells were monitored by fluorescent microscopy after cell staining with Hoechst 33342 and ethydium bromide.

**Results:**

Half-maximal inhibitory concentration (IC_50_) of Les-3288, Dox, and TMZ was calculated for human glioblastoma U251 cells. The rating of the values of this indicator of cellular vitality was assessed. The results of MTT assay proved the superiority of Les-3288 vs Les-3288>Dox>TMZ, which is in agreement with the results of Trypan blue testing showing Les-3288 ≈ Dox>TMZ. In general, such ranking corresponded to a scale of pro-apoptotic impairments in the morphology of glioma U251 cells and the results of Western-blot analysis of cleaved Caspase 3. Contrary to Dox, Les-3288 and TMZ did not affect significantly ROS levels in the treated cells.

**Conclusion:**

The effect of the synthetic 4-thiazolidinone derivative Les-3288 is realized via apoptosis mechanisms and does not involve ROS. In comparison with Dox and TMZ, it is more effective in destroying human glioblastoma U251 cells. Les-3288 compound has a potential as an anticancer drug for glioblastoma. Nevertheless, further preclinical studies of the blood-brain barrier are needed.

There are approximately 86 billion neurons and roughly same number of glial cells in human brain and spinal cord (1,2). Gliomas are primary brain tumors known to be the most aggressive (2-4) and developing by the malignant transformation of astrocytes (5). There are three main reasons why gliomas are still so difficult to treat: a necessity for anticancer drug to circumvent the blood-brain barrier; poor response of tumor to chemotherapy; rapid development of resistance of glioma cells to applied anticancer drugs (6).

Due to its invasiveness, elective and definite removal of glioblastoma in adults is almost impossible (7). In the oncology practice, Temozolomide (TMZ), an alkylating medicine, is used for standard second-line chemotherapy of astrocytoma and first-line chemotherapy of glioblastoma multiforme (8,9). The median survival time of treated patients with glioblastoma is no longer than 12-15 months, and the main reason is drug resistance. In future, circumvention of TMZ resistance can improve treatment outcomes. The principal ways of overcoming chemo-resistance are the following: 1) to increase the efflux of chemotherapeutic drugs (10); 2) to induce an expression of anti-apoptotic proteins (4,11,12); 3) to activated DNA repair pathways (13-15). A search for novel chemotherapy for glioblastoma that is more effective than TMZ is still actual and necessary.

Recently, a big collection of anti-glioblastoma drugs possessing different mechanisms of action was considered by the National Institute of Health. 446 such drugs approved by the FDA were under screening aimed at revealing the most perspective new therapeutics for clinical trials. Among 22 most potent anti-glioblastoma drugs (death of >50% cells) were not only traditional anticancer agents, but also blockers of serotonin, statins that lower cholesterol level, as well as some anti-inflammatory drugs and the modulators of hormonal activity (16).

The antineoplastic effect of novel 4-thiazolidinone derivative denoted as Les-3288 was studied on 60 human tumor cell lines by the National Cancer Institute (USA) (17,18). It was found that CNS human cancer cells of SF-539 line were the most sensitive to the action of Les-3288 with a positive cytostatic effect in 4 of 60 tumor cell lines and a positive cytotoxic effect in 56 of 60 tumor cell lines. While human melanoma cells of SK-MEL-5 line were the most sensitive to the action of Les-3833 with a positive cytostatic effect in 0 of 59 tumor cell lines and a positive cytotoxic effect in 59 of 59 tumor cell lines (17,18).

Dox is used to treat many types of cancer, including leukemia, lymphoma, neuroblastoma, sarcoma, Wilms tumor, and cancers of the lung, breast, stomach, ovary, thyroid, and bladder cancer. Dox inhibits the activity of the enzyme topoisomerase II which relaxes supercoils in DNA for transcription (19,20). TMZ (Temodar) is an alkylating agent forming a molecular bond in the DNA strands inside tumor cells preventing their successful replication (21). Based on the literature, the thiazolidines conjugates have anticancer activity via their affinity to tyrosine kinase, cyclin-dependent kinases and carbonic anhydrase isozymes (22).

Non-traditional anti-cancer drugs could be applied as a second-line medicine for chemotherapy of glioblastomas. The statins were shown to decrease glioblastoma cell proliferation *in vitro* and induce their autophagy (16). It should be noted that they also increased significantly the induction of apoptosis caused by a known topoisomerase I inhibitor, irinotecan, that is applied in the chemotherapeutic schemes for treatment of glioblastomas, as well as many other cancers, mainly as a first-line medicine for colorectal cancers and tumors of the gastrointestinal tract (16,23). In patients with a malignant glioma, the irinotecan was used as a monotherapy or combined chemotherapy together with TMZ, carmustine, thalidomide, or bevacizumab (23). It was suggested that statins prevent glycosylation of the MDR-1 protein that is important in the multi-drug resistance mechanisms.

The efforts were performed to establish a regularity of the capability of different compounds to cross BBB by passive diffusion (24). It was found that there are three main characteristics that define such capability: 1) air-water partition coefficient (Kaw); 2) critical micelle concentration (CMCD); 3) cross-sectional area (AD). According to these parameters, the Les-3288 could cross the BBB. However, to answer this question more definitely, special studies at the morphological level using brain cells of the microvascular endothelium or at the molecular level using the the lipid bilayer as a diffusion barrier (24) should be carried out.

In previously published paper, we addressed further characterization of the action of potential anticancer drug Les-3288 (25). The cytotoxic effects of the Les-3288 were promising in comparison to anticancer drugs as TMZ and Dox. In this study, we compared their pro-apoptotic effects of in the treatment of human glioma U251 cells to improve treatment outcomes of glioblastoma and avoid anticancer drug resistance.

## METHODS

### Drugs

Doxorubicin (Pfizer, Italy) and Temozolomide (Merck, Germany) were used in this study. The Les-3288 compound was synthesized at the Department of Pharmaceutical, Organic and Bioorganic Chemistry of Danylo Halytsky Lviv National Medical University, as described earlier (18,26). Before use, it was dissolved in the dimethylsulfoxide (DMSO, Arterium, Ukraine), and then additionally dissolved in distilled water. The final concentration of the DMSO in the cultural medium did not exceed 0.1%.

### Cell culture

Human glioma cells of U251 line were obtained from the Collection of cell cultures at the Institute of Molecular Biology and Genetics, National Academy of Science of Ukraine (Kyiv, Ukraine). The cells were cultured in Dulbecco's modiﬁed Eagle's medium (DMEM, Sigma, USA) supplemented with 10% fetal bovine serum (Sigma, USA). Cells were grown in the CO_2_-thermostate at 37°C, 5% CO_2_ and 95% humidity. The reseeding of cells was performed at 1:5 ratio once in 2-3 days.

### Evaluation of cytotoxic action of studied substances

Cells were plated in 96- or 24-well plastic plates (Greiner bio-one, USA), and substances were added after 24 h of cell seeding. Cell number was counted in the hemocytometric chamber after staining with Trypan Blue dye (DV-T10282, Invitrogen, Life Technologies Corporation) at 0.04% final concentration. The dead cells with damaged plasma membrane were stained with Trypan Blue due to the uptake of this dye.

A part of viable cells after drug treatment was determined by the MTT (3-[4,5-dimethylthiazol-2-yl]-2,5-diphenyltetrazolium bromide; thiazolyl blue) assay, as recommended by the manufacturer (Sigma, USA). Purple product of the reaction (formazan crystals dissolved in the DMSO) was quantitatively measured in a multi-channel microphotometer BioTek 76 883 (BioTek, USA) at 620 nm wavelength.

### Light and fluorescence microscopy

The living, apoptotic, and necrotic cells were observed under the inverted light microscope Biolam (LOMO, Russian Federation). Human glioma U251 cells were seeded on glass microscopic slides in the 24-well plates (Greiner bio-one, USA), and substances were added 24 h and 48 h. Chromatin material of cell nucleus living and apoptosis cells was stained with the DNA-specific fluorescent dye Hoechst 33342 (Sigma, USA). Cells were also stained with the DNA/RNA-specific fluorescent dye Ethidium bromide (Sigma, USA). The dead cells uptake this dye due to a damage of their plasma membrane. These fluorochromes were added to cultured cells in the following final concentrations: Hoechst 33342 – 0.2-0.5 µg/mL, and cells were incubated for 20-30 min, Ethidium bromide – 0.1 µg/mL, was added immediately before viewing and photographing.Images were processed with a fluorescent Zeiss microscope (Carl Zeiss, Germany) using AxioImager A1 camera ( ~ 400X magnification).

### Western-blot analysis

After 48 h exposure of cells to tested compound, cellular proteins were isolated, resolved by the SDS/PAGE, and transferred onto a poly-vinylidenedifluoride (PVDF) membrane for the Western-blot analysis, as described (27). Anti-cleaved Caspase 3 (Cell Signaling Technology, USA) and anti-p-p MAPK 42/44 antibodies were used at a 1:1000 dilution. Equal loading of protein on each lane was evaluated by the immunoblotting of the same membrane with anti-beta-actin monoclonal mouse AC-15 (Sigma-Aldrich, USA). All secondary antibodies were peroxidase-labeled (Cell Signaling, USA) and used at working dilution of 1:5,000.

### Reactive oxygen species (ROS) measurement

For ROS measurement, mean cell fluorescence was analyzed on FL1 channel of FACScan flow cytometer (BD Biosciences, Mountain View, CA). ROS content in cells was measured by their staining with fluorescent dihydroethidum (DHE, O_2_–specific dye) after incubating the control (untreated) or drug-treated (6 h, 12h , 24 h) cells. 3x10^5^ glioma U251 cells were pre-incubated with the DHE (10 μM) for 30 min at 37°C before their measurement at FL2 channel of the FACSCalibur flow cytometer (BD Biosciences, San Jose, CA, USA).

### Data analysis and statistics

All experiments were repeated three times with three parallels in each variant. The Analysis of Variance (ANOVA) was used as statistics test for comparison of experimental groups. 2-way ANOVA with Bonferroni post-tests in order to compare replicate means by rows was applied using GraphPad Prism v6.0 software. The results are presented as a mean ± SD by GraphPad Prism 6.0 program, *P* < 0.05 was considered as statistically significant.

## RESULTS

After the human glioma U251 cells were treated with 4-thiazolidinone derivative Les-3288, Dox and TMZ, the viability and death of the glioma cells were studied using MTT assay and Trypan blue exclusion test, correspondingly. Western-blot analysis of proteins of glioma U251 cells was performed, and fluorescent dyes and fluorescence microscopy were used for the detection and evaluation of cytomorphological changes. Pro-apoptotic products in cultured cells were evaluated, and fluorescence-activated cell sorting (FACS) analysis was applied to determine the ROS level in the treated cells.

### MTT assay of cell viability and Trypan blue exclusion testing of cytotoxic effect of 4-thiazolidinone derivative Les-3288, Doxorubicin and Temozolomide

**Figure 1:**
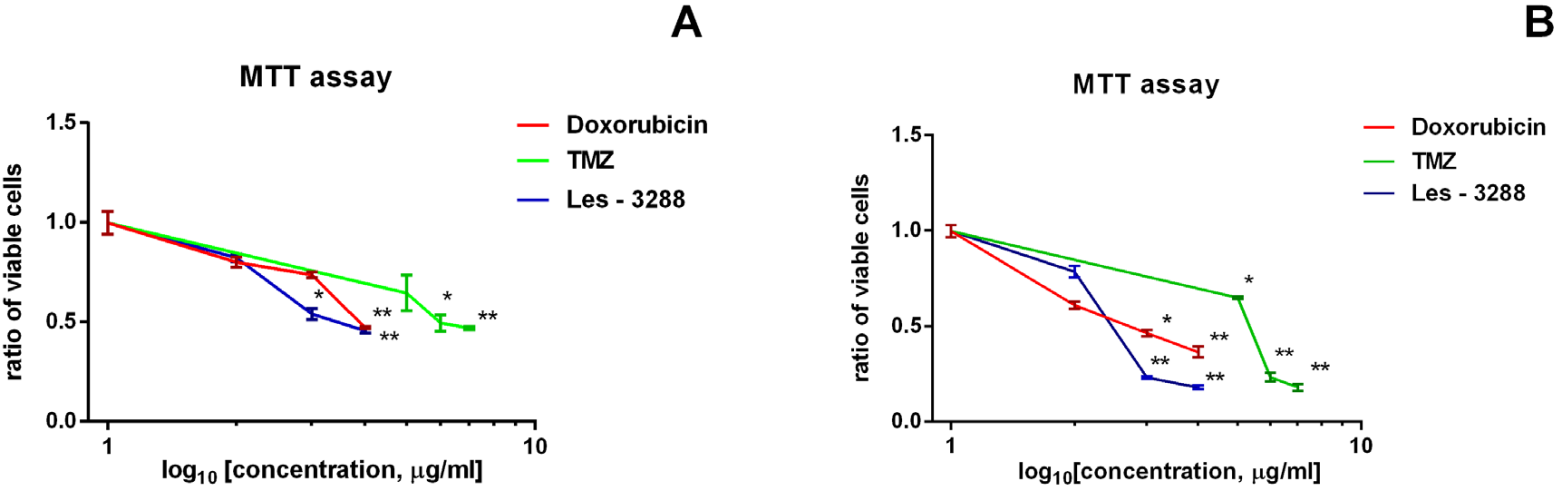
Number of alive human glioma U251 cells under treatment for 24 h and 48 h with Les-3288, doxorubicine (Dox), and temozolomide (TMZ). Negative control – 100% (untreated cells cultured for 24 h and 48 h).

The results of MTT assay demonstrated that the Les-3288 compound was more potent than Dox and TMZ in cell inhibiting activity er during 24 h treatment, although Dox demonstrated higher capability of inhibiting cellular viability after 48 h treatment. The TMZ showed weak cytotoxicity only at high doses (10 µg/mL) and at durable treatment (48 h) (Figure 1).

**Figure 2:**
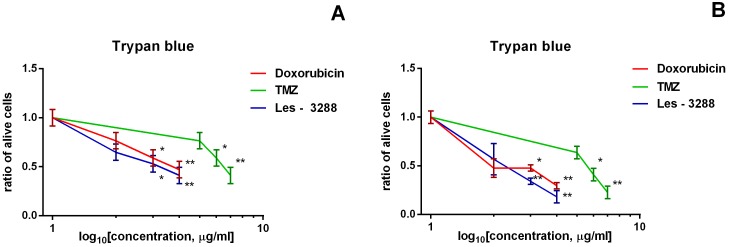
Results of Trypan blue testing of death of human glioma U251 cells treated with the Les-3288, doxorubicine (Dox), and temozolomide (TMZ). Negative control – 100% (untreated cells cultured for 24 h and 48 h).

The results of evaluating cytotoxic action of Les-3288, Dox, and TMZ by using Trypan blue exclusion test confirmed the results of MTT assay measuring cell viability (Figure 2). The Les-3288 demonstrated similar toxicity toward human glioma cells of U251 line, as the Dox did at 24 and 48 h, while the TMZ was relatively non-toxic even in high doses (5 and 10 µg/mL) used in the experiments in both time points of measurement.

The values of 50% inhibition concentration (IC_50_) were determined by the MTT assay and 50% lethal concentration (LC_50_) were determined by the Trypan blue exclusion test of glioma cells treated for 24 and 48 h with the Les-3288, Dox, and TMZ. Cytotoxic effects of studied agents demonstrated the highest cytotoxic effect of the Les-3288 (Table 1). These results of studying human glioma U251 cells agree with the results of our previous investigation, which showed that the Les-3288 was the most toxic for rat glioma C6 cells among the similar compounds (Les-3882 and Les-3833) and Dox (25).

**Table 1:** Half-maximal inhibitory concentration (IC_50_) determined in the cell viability and proliferation assay (MTT) assay and Trypan blue exclusion testing of human glioblastoma U251 cells treated for 24 h and 48 h with the Les-3288, doxorubicine (Dox), and temozolomide (TMZ)

IC_50_ μg/mL	MTT assay		Trypan blue testing	
	24 h	48 h	24 h	48 h
**Dox**	0.96	0.41	0.88	0.13
**Les-3288**	0.74	0.31	0.63	0.22
**TMZ**	50.26	24.43	50.02	34.00

### Western-blot analysis of apoptosis-related proteins in human glioma U251 cells treated with 4-thiazolidinone derivative Les-3288, doxorubicin and temozolomide

**Figure 3:**
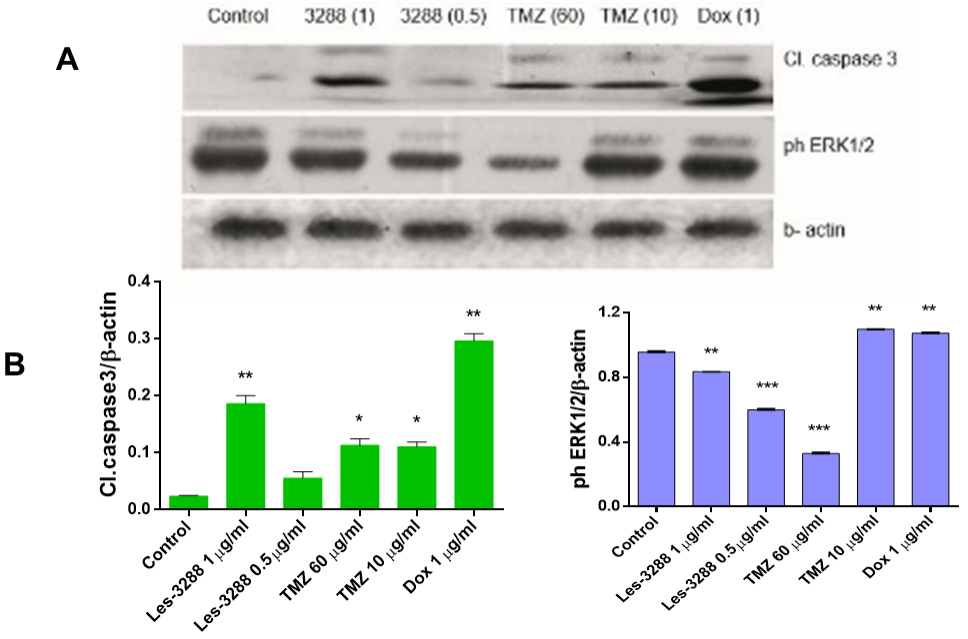
The results of Western-blot (**A**) and densitometry (**B**) analyses of the apoptosis-related proteins – cleaved caspase 3 and ERK1/2-kinase – in human glioma U251 cells treated for 24 h with the Les-3288, doxorubicine (Dox), and temozolomide (TMZ). *P ≤ 0.05; **P ≤ 0.01, ***P ≤ 0.001 (difference compared with the control).

The results of Western-blot analysis of proteins of human glioma U251 cells treated with 4-thiazolidinone derivative Les-3288, Dox and TMZ showed different character of action of Les-3288 and TMZ in relation to cleaved Caspase-3 that is a classical bio-marker of apoptosis (28). An elevation in the Les-3288 dose from 0.5 to 1.0 µg/mL led to an increase in the amount of the cleaved Caspase-3 in treated glioma U251 cells, while the effect of TMZ did not change with increasing its dose from 10 and 60 µg/mL (Figure 3). These results were confirmed by the densitometry analysis, also showing the strongest effect of Dox in 1.0 µg/mL dose (Figure 3). ERK1/2-kinase is known be involved in cellular response to various external stressing agents (29). Cell treatment with Les-3288 led to a dose-dependent increase in ERK1/2-kinase, while TMZ dose-dependently decreased the level of this kinase, and Dox did not have any effect (Figure 3).

### Characteristics of morphological changes in human glioma U251 cells treated with 4-thiazolidinone derivative Les-3288, doxorubicin and temozolomide

**Figure 4:**
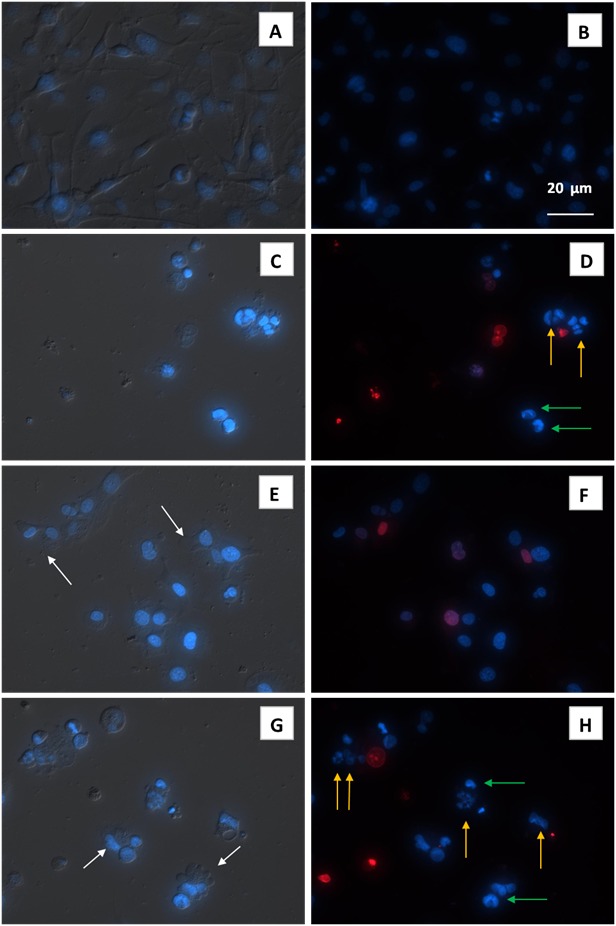
Cytomorphological characteristics (fluorescence microscopy) of human glioma U251 cells treated for 48 h with 4-thiazolidinone derivative Les-3288 (E, F), doxorubicine (Dox) (C, D), and temozolomide (TMZ) (G, H); control – (A, B). Left – DIC image of treated cells. Les-3288 and Dox were used in 1 ug/mL dose, while TMZ was used in 10 µg/mL dose. Right – fluorescent image of treated cells (blue color – staining with fluorescent DNA-specific dye Hoechst-33342, red color – staining of damaged cells with the ethydium bromide. White arrows - plasma membrane blebbing, green arrows - condensed chromatin, yellow arrows - nucleus fragmentation.

The morphological changes in the treated cells were evaluated using fluorescent dyes Hoechst 33342 and ethidium bromide (Figure 4). The treatment of cells with studies agents led to an appearance of cells with changed morphology of the nucleus (condensed chromatin - green arrow, fragmentation of nucleus - yellow arrow) and formation of plasma membrane vesicles (white arrow). It was rather complicated to quantify the cytotoxicity level considering the results of the morphological (microscopic) studies. Nevertheless, in control (untreated cells, Figure 4 A and B) there were no cells with a condensed chromatin and membrane damage that could detected as accumulation of red fluorescence of the Ethidium bromide inside the cells. (Figure 4 D-H)

The microscopic study of the morphological characteristics of human glioma U251 cells treated with Les-3288, Dox, and TMZ demonstrated that the Dox and Les-3288 used in 1 µg/mL dose caused the most drastic changes in cell intactness, while the action of the TMZ (10 µg/mL) was much less effective. We observed a decrease in cell number and their size due to the induction of apoptosis. Besides, cell debris appeared under the action of Dox and Les-3288 on the glioma cells. We also observed conglomerates of damaged (dead or dying) cells surrounded by the remains of the destroyed cells. Fragments of the apoptotic cell nuclei were also observed (Figure 4 C and D). The Les-3288 caused less drastic changes in the glioma cells, comparing with that induced by the Dox. Les-3288 caused a decrease in number of these cells, although there was an increased number of cells with condensed chromatin, as well as cells with red fluorescence of the Ethidium bromide due to an impaired plasma membrane (Figure 4 E and F). The morphological changes in the glioma cells induced by the TMZ were less expressed than those caused by the Dox and Les-3288. In general, TMZ-treated cells looked more round and less damaged (Figure 4 G and H).

Thus, the results of the microscopic study of the morphological characteristics of human glioma U251 cells treated with substances under study were in accordance with the results of the MTT assay of the action of Dox and Les-3288 used in 1 µg/mL dose.

### FACS analysis of ROS level human glioma U251 cells treated with the 4-thiazolidinone derivative Les-3288, doxorubicin and temozolomide

**Figure 5:**
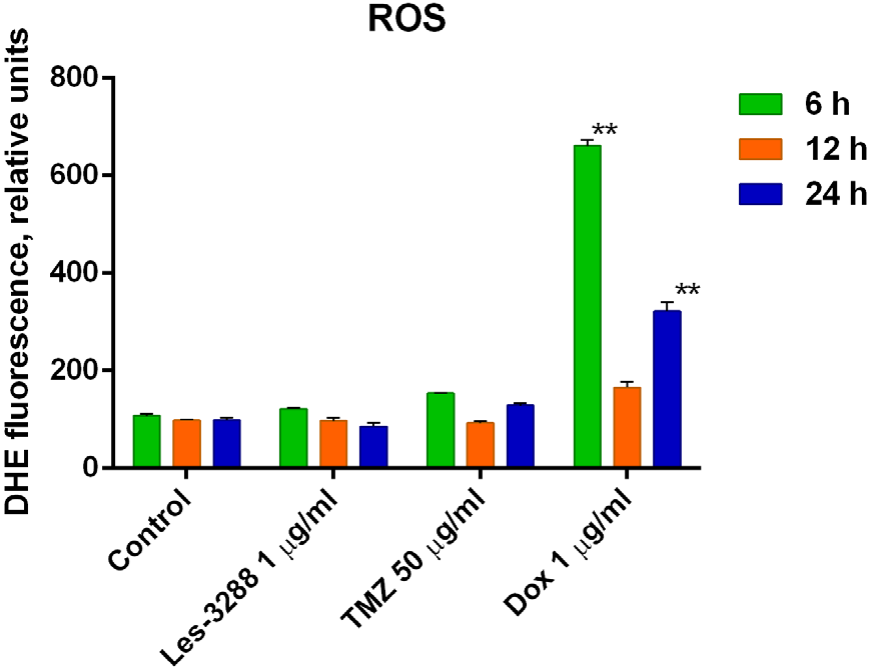
The results of fluorescence-activated cell sorting (FACS) analysis of reactive oxygen species (ROS) level human glioma U251 cells treated with the 4-thiazolidinone derivative Les-3288, doxorubicine (Dox), and temozolomide (TMZ). *P ≤ 0.05; **P ≤ 0.01, ***P ≤ 0.001 (difference compared with the control).

ROS level measured by the dihydroethidium (DHE) staining was not changed in the glioma cells treated for 6-24 h with the Les-3288 or the TMZ. Opposite to that, Dox induced relatively rapid (6 h) increase in ROS level that was diminished during further (12-24 h) treatment of cells (Figure 5).

## DISCUSSION

In this study, we compared various pro-apoptotic effects in human glioma cells of U251 line of novel potential anti-glioma agent – 4-thiazolidinone derivative Les-3288 (25), TMZ that is a principal medication used for glioma treatment (30,31), and Dox that is known to be the “golden standard” in cancer chemotherapy (2). In previous study, it was shown that the 4-thiazolidinone derivatives induced apoptosis in mammalian leukemia cells using mitochondria-depended pathway as the main mode of their action. These compounds also induced the G0/G1 arrest of the treated cells and caused inhibition of cell division (32).

Recently, we have shown that TMZ caused apoptosis in human glioma cells of T98G line and rat glioma cells of C6 line via activation of MAPK signaling pathway and inhibition of STAT3, and stopped glioma cells in G2/M phase of cell cycle (33). In another study, we have demonstrated the pro-apoptotic action of the Les-3288 compound toward rat glioma cells of C6 line that possessed even higher toxicity for these cells than that of the Dox (25). In present study, we addressed the mechanisms of the pro-apoptotic action of the compound Les-3288, TMZ and Dox in order to reveal potential differences in these mechanisms and, thus, consider them at planning the combined chemotherapy schemes for glioma treatment.

The Les-3288 compound demonstrated a cytotoxic action comparable with that of the Dox, while the TMZ, if judged by the value of the half-maximal inhibitory concentration (IC_50_), was approximately 10 times less toxic for human glioma U251 cells that of two other studied agents – Les-3288 and Dox. The results of the MTT measuring of the IC_50_ at the action of the compared agents demonstrated the following rank of decreasing cytotoxicity – Les-3288>Dox>TMZ. Trypan blue exclusion assay of the treated glioma cells ranked these agents in: Les-3288 ≈ Dox>TMZ. It should be noted that the character of these cytotoxicity ranks did not differ significantly at 24 to 48 h duration of glioma cell treatment.

The Western-blot analysis was used for estimating the amount of apoptosis-related protein, caspase 3, and cell stress-responsive protein kinase – Erk1/2 (10). The results of the Western-blot analysis confirmed our data on the cytotoxicity of the Les-3288, Dox, and TMZ. Caspase 3 is an effector enzyme that plays a central role in the pro-apoptotic proteolytic destruction of principle intracellular regulatory proteins of cell cycling (10) and DNA reparation (29). As predicted, the level of caspase 3 was induced in human glioma U251 cells by the Les-3288 and TMZ, and Dox action was the strongest there.

The uniqueness of the ERK1/2 is based on their bi-functionality, since they participate in controlling both cell proliferation and apoptosis (34). If the role of ERK1/2 in cell proliferation can be easily explained, the mechanisms of their action in cell death are less understood. Activation of ERK1/2 can lead to tumor development through phosphorylation of Bim and Bid (promoters of apoptosis) that causes proteosomal degradation of these proteins, thus, blocking apoptosis (34). However, DNA damaging agents, namely some anticancer drugs or x-ray- and UV-irradiation activate ERK1/2 that leads to apoptosis of targeted cells (35).

While activation of ERK caused by low grade DNA damage leads to arrest of cell cycle, activation of this kinase caused by a significant damage of DNA leads to apoptosis (36). Cisplatin and Dox that are damaging DNA and inducing ROS generation also activate ERK (37,38), and inhibition of ERK blocks apoptosis (39).

Thus, using ERK1/2 response as an indicator of cytotoxicity could be risky. It might demonstrate the presence of stressing action, however, it cannot show uniformly a direction of cell response to that action. In our case, the ID3288 compound dose-dependently stimulated Erk1/2 in human glioma cells of U251 line, while the temozolomide acted oppositely dose-dependently inhibiting this kinase, and doxorubicin did not affect it significantly (29,34-36).

The conclusion could be done here that all three agents under study, probably, use different mechanisms of the inhibition of cell proliferation and induction of apoptosis in glioma cells.

FACS analysis showed that neither TMZ, nor Les-3288 induced a significant production of ROS in glioma cells, comparing with such action of the Dox. These results on ROS level in drug-treated glioma cells *in vitro* agree with our former data on measuring ROS level in blood serum of rats treated with the same drugs, namely the Les-3288 and Dox (25).

In conclusion, we showed that a novel synthetic 4-thiazolidinone derivative Les-3288 possesses highly toxic action toward human glioma U251 cells. Therefore, Les-3288 has a potential in human use to overcome chemo-resistance. It was found that its anticancer effect is at the same level as doxorubicin, and approximately 10 times (regarding IC_50_) more pronounced than such action of the Temozolomide. Thus, the Les-3288 could be a good candidate for the chemotherapy of glioblastoma, since it is more effective than the TMZ currently used in clinical practice.

It is important that anti-glioma drugs can cross blood-brain barrier (BBB). Some anticancer drugs are known to pass through the BBB (procarbazine, temozolomide (Temodar), methotrexate) (21). Dox does not cross BBB. Following its intravenous administration about 35% of the drug is bound to plasma proteins and, thus, could not cross the BBB. Taking into account the intermediate hydrophobicity of Les-3288 and its molecular mass (559.44 g/mol), this compound has a chance to cross the BBB (24).

To address these perspectives, we have started experiments in vitro applying a combined treatment of human glioma cells with the 4-thiazolidinone derivative Les-3288 and temozolomide. In the ongoing translational research, we are progressing with the formation of water-soluble form of the Les-3288. An investigation of the bio-distribution, including the capability to cross blood-brain barrier, is on the way. We are also working on the biocompatible and efficient drug delivery systems with magnetic iron nanoparticles suitable for brain MRI to be able to locate gliomas, define the tumor borderlines, and treat certain brain tumors.
